# Chromosome Condensation 1-Like (*Chc1L*) Is a Novel Tumor Suppressor Involved in Development of Histiocyte-Rich Neoplasms

**DOI:** 10.1371/journal.pone.0135755

**Published:** 2015-08-20

**Authors:** David R. Spillane, Ding Yan Wang, Susan Newbigging, Youdong Wang, Chang-Xin Shi, Hae-Ra Cho, Hiroki Shimizu, Anthony Gramolini, Mingyao Liu, Xiao-Yan Wen

**Affiliations:** 1 Keenan Research Centre for Biomedical Science, Li Ka Shing Knowledge Institute, St. Michael’s Hospital, Toronto, Ontario, Canada; 2 Department of Medicine & Institute of Medical Science, University of Toronto, Toronto, Ontario, Canada; 3 Department of Physiology, University of Toronto, Toronto, Ontario, Canada; 4 Centre for Modeling Human Disease, Samuel Lunenfeld Research Institute, Mount Sinai Hospital, The Toronto Centre for Phenogenomics, University of Toronto, Toronto, Ontario, Canada; 5 Latner Thoracic Surgery Research Laboratories, Toronto General Research Institute, University Health Network, Toronto, Ontario, Canada; University of Navarra, SPAIN

## Abstract

Human chromosomal region 13q14 is a deletion hotspot in prostate cancer, multiple myeloma, and chronic lymphocytic leukemia. This region is believed to host multiple tumor suppressors. Chromosome Condensation 1-like (*CHC1L*) is located at 13q14, and found within the smallest common region of loss of heterozygosity in prostate cancer. Decreased expression of *CHC1L* is linked to pathogenesis and progression of both prostate cancer and multiple myeloma. However, there is no direct evidence for *CHC1L*’s putative tumor suppressing role in current literature. Presently, we describe the generation and characterization of *Chc1L* knockout mice. *Chc1L* ^-/-^ mice do not develop cancer at a young age, but bone marrow and spleen cells from 8–12 week-old mice display an exaggerated proliferative response. By approximately two years of age, knockout and heterozygote mice have a markedly increased incidence of tumorigenesis compared to wild-type controls, with tumors occurring mainly in the spleen, mesenteric lymph nodes, liver and intestinal tract. Histopathological analysis found that most heterozygote and knockout mice succumb to either Histiocytic Sarcoma or Histiocyte-Associated Lymphoma. Our study suggests that *Chc1L* is involved in suppression of these two histiocyte-rich neoplasms in mice and supports clinical data suggesting that *CHC1L* loss of function is an important step in the pathogenesis of cancers containing 13q14 deletion.

## Introduction

Chromosome condensation 1-like (*CHC1L*, aka *RCBTB2*) is an uncharacterized gene residing at 13q14.3 in humans. This region is frequently lost in several human cancers, including B cell chronic lymphocytic leukemia (CLL) (70% incidence of deletion [1]), multiple myeloma (MM) (40–50% incidence [2]), and prostate cancer (PC) (33% incidence [3]), as well as myeloid disorders and dendritic sarcomas [4, 5]. The high frequency of deletion suggests the existence of tumor suppressor genes residing in this region.

In an effort to identify candidate tumor suppressors, several groups have mapped 13q14 deletions [6, 7]. *CHC1L* is located within the smallest common region of loss of heterozygosity (LOH) in PC [8]. Expression is decreased at least 2-fold in 58% of all PC tumors, as well as in the three prostate cancer cell lines LNCaP, DU145, and PC3 [8]. Among PC tumors with LOH at 13q14, *CHC1L* is significantly down-regulated in 78% of cases [8]. Additionally, low expression levels of *CHC1L* are frequently observed in MM patients [9].

The murine orthologue encodes a protein of 551 amino acids, sharing 95% identity with human CHC1L. Mouse studies have proposed a role during acrosome formation in developing spermatocytes through regulation of nuclear transport [10]. CHC1L possesses RCC1-like repeats on its N-terminal and BTB domains on its C-terminal [11].

While previous studies have shown an association between cancer occurrence and *CHC1L* deletion/under-expression, a reverse genetic approach is needed to show the contribution of loss of function to tumorigenesis in order to validate its hypothesized tumor suppressive function. Here, we describe the generation and characterization of *Chc1L* knockout mice.

## Materials and Methods

### Experimental animals

Experimental mice were generated on a C57/BL6 background. The protocol was approved by the Ethics Board of the Animal Resource Center at Princess Margaret Hospital (Toronto, ON) (Protocol ACC418), where the animals were housed. The Animal Resource Center is fully accredited by the Canadian Council for Animal Care. Animals were maintained on standard feed and water *ad libitum*, in a clean, temperature-controlled environment with standard day-light cycles.

To minimize stress, animals were euthanized in their home cages using carbon dioxide, followed by cervical dislocation as secondary euthanasia. All efforts were made to minimize suffering. Dissections were performed by an experience technician and graduate students in a biosafety cabinet.

### Gene targeting strategy

**Fig 1:**
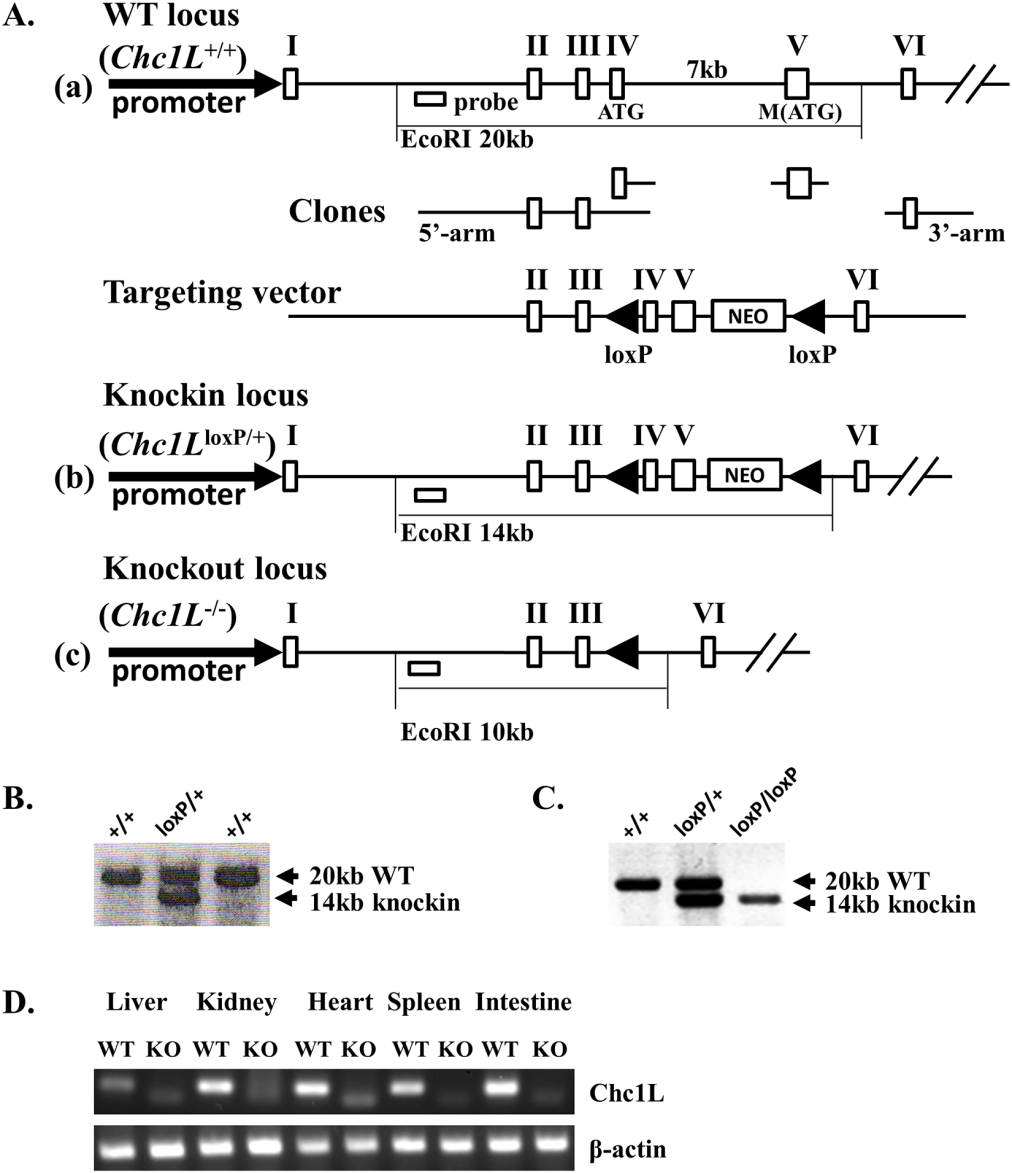
Generation of *Chc1L* ^-/-^ knockout mice by gene targeting. A) Gene targeting strategy. (a) The start codon is located in exon 4 of murine *Chc1L*, and a second ATG codon is in exon 5. EcoRI digestion produces a gene fragment of 20 kb. Deleting 6kb of intron 4, a gene targeting vector was constructed, with unidirectional loxP sites flanking exons 4 and 5. (b) The gene-targeted locus produces a 14 kb fragment upon digestion with EcoRI. (c) Exon 4, intron 4 and exon 5 are lost following Cre-mediated recombination. B) Confirmation of gene targeting in ES cells. Successful knockin of neomycin-selected ES cells was detected by Southern Blot using a 5’ probe, visualizing a 20kb WT locus fragment, and a 14kb gene-targeted fragment. C) Confirmation of gene targeting in mice. Gene knockin was confirmed in mice again using Southern Blot. D) Confirmation of loss of Chc1L expression. Loss of Chc1L expression was confirmed by RT-PCR.

Murine *Chc1L* contains a start codon in exon 4, and a second ATG in exon 5. In order to prevent the second ATG from assuming start codon activity, both exons were flanked by unidirectional loxP sites (Fig 1A). ES cells were electroporated with the targeting vector and selected by neomycin resistance. Successful knockin was confirmed by Southern Blot (Fig 1B).

Gene targeted ES cells were injected into embryos which were implanted into pseudopregnant mice. Gene targeting in the progeny was confirmed by Southern Blot (Fig 1C). *Chc1L* ^*loxP*/+^ mice were then crossed to a line expressing Cre recombinase under the *Blimp1* promoter, active in germ cells [12, 13]. The F2 generations possessed nonconditional deletion of exons 4 and 5.

### Genotyping strategy

Tissue was incubated overnight in lysis buffer at 55°C (1.5 mM MgCl2, 50 mM KCl, 10 mM Tris-HCl pH 8.3, 20 μg/mL protease K). Intron 4 primers detected the wild-type allele (Int4F 5’- GTGTTACTTTTGCCCGTGGT-3’, and Int4R 5’- GTGACAGGGCAAACCAAGTT-3’). Introns 3 and 5 are brought into close enough proximity to be amplified in the knockout but not wild-type tissues (DelF 5’- TTCGCTACCTTAGGACCGTTA-3’, DelR 5’- GGGTACCGAATTCCTCGAC 3’).

### RT-PCR

Verification of knockout at the protein level was difficult due to a lack of specificity among commercially available antibodies. Therefore, RT-PCR was used. Tissues were collected in RNase-free conditions and snap frozen. RNA was collected using QIAGEN RNeasy Plus kit (Qiagen, Cat. No. 74134; Venlo, Limburg, Netherlands) according to manufacturer’s protocol. RNA integrity was verified on an Agilent 2100 Bioanalyzer (Santa Clara, CA, USA). RNA was reverse transcribed using Applied Biosystems’ High Capacity cDNA Reverse Transcription Kit (Applied Biosystems, 4368814; Carlsbad, CA, USA). Two sets of primers were used for detection of the wild-type transcript by amplifying around the exon 4/5 splice site (F1 5’-AGGGACTGCACAGGACTGAT-3’, R1 5’- CAAGCCTGACGAATCAACTG-3’ and F2 5’- TGGAAGAAGAAGTGCCTGGT-3’, R2 5’-GGCCACTTTCCCACATCTAA-3’). The identity of the amplicon was confirmed by sequencing.

### MTT assay

Cells were isolated from age- and sex-matched mice between 8–12 weeks of age. Bone marrow and spleen cells were cultured in triplicate at 2x105 cells per well in a 96-well plate with 200 μL DMEM/10%FBS/1%Antibiotic + LPS for 48 hours. 10 μL MTT solution (5 mg/mL MTT (Invitrogen, catalogue number M-6494; Carlsbad, CA, USA) in PBS) was added to each well, and cultured for 4 hours. 100μL solubilisation solution (10% SDS in ddH20+100 μL of 37% HCl per 100 mL) was added to each well, and cultured for 4 hours. OD was read at 570 nm. Experiment was performed on three pairs of animals.

### Histology

All tissues were collected into formalin and fixed overnight. Tissues were embedded in paraffin, sectioned, and stained by hematoxylin and eosin. For immunohistochemistry (IHC), standard tissue sections were deparaffinized, rehydrated and post-fixed in 10% neutral-buffered formalin for 1 hour. Sections were soaked in 10mM citrate buffer (pH 6.0) at 85°C for 3 hours (for Mac-2 only). Slides were air-dried and washed in PBST, then soaked in 3% hydrogen peroxide in methanol for 30 minutes. Slides were blocked using Dako protein block (Dako: catalogue number X0909; Santa Clara, CA, USA). 1:100 diluted monoclonal rat anti-Mac-2 (Cedarlane: catalogue number CL8942AP; Burlington, ON, CA), rat monoclonal anti-F4/80 (ABCAM: catalogue number AB6640; Cambridge, England, UK) or rat monoclonal anti-B220 (BD Bioscience: catalogue number 550286; Franklin Lakes, NJ, USA) was pipetted onto slide surface and incubated overnight at 4°C. The slides were washed and incubated in 1:100 anti-rat IgG-biotin (Vector Labs: catalog number BA-4001; Burlingame, CA, USA) at room temperature for 60 minutes. A and B from kit ABC (Vector Labs: catalogue number PK-6100; Burlingame, CA, USA) were pipetted onto slides, and incubated 30 minutes room temperature. Slides were immersed in DAB colour development solution, then washed in distilled water. Slides were counterstained with hematoxylin, dipped in 1% acid alcohol, and then immersed in Scott’s tap water. Finally, slides were dehydrated and cleared, then coverslipped with Permount.

### Statistical methods

P values for tumor incidence were calculated using the Chi-squared test with a 3x2 contingency table.

P values for MTT cell survival were calculated using the two-tailed student’s T test.

## Results

### Chc1L-deficiency increases incidence of tumorigenesis

To examine the role of *Chc1L* in tumorigenesis, we knocked out the murine *Chc1L* gene by germ-line deletion of the ATG-containing exons 4 and 5 through Cre-mediated recombination (Fig 1A). Successful gene targeting in ES cells and knockout mice was confirmed by Southern Blotting (Fig 1B and 1C), and loss of expression was validated by RT-PCR and sequencing (Fig 1D). *Chc1L* ^+/+^, *Chc1L* ^+/-^ and *Chc1L* ^-/-^ mice were born at Mendelian frequency, indicating that they have viable embryonic development.

Histopathological analysis of spleen, lymph node and liver tissue was performed with tissues isolated from *Chc1L* ^-/-^ mice aged 8–12 weeks. No visible general pathology was observed, and there was no evidence of early neoplastic events.

**Fig 2:**
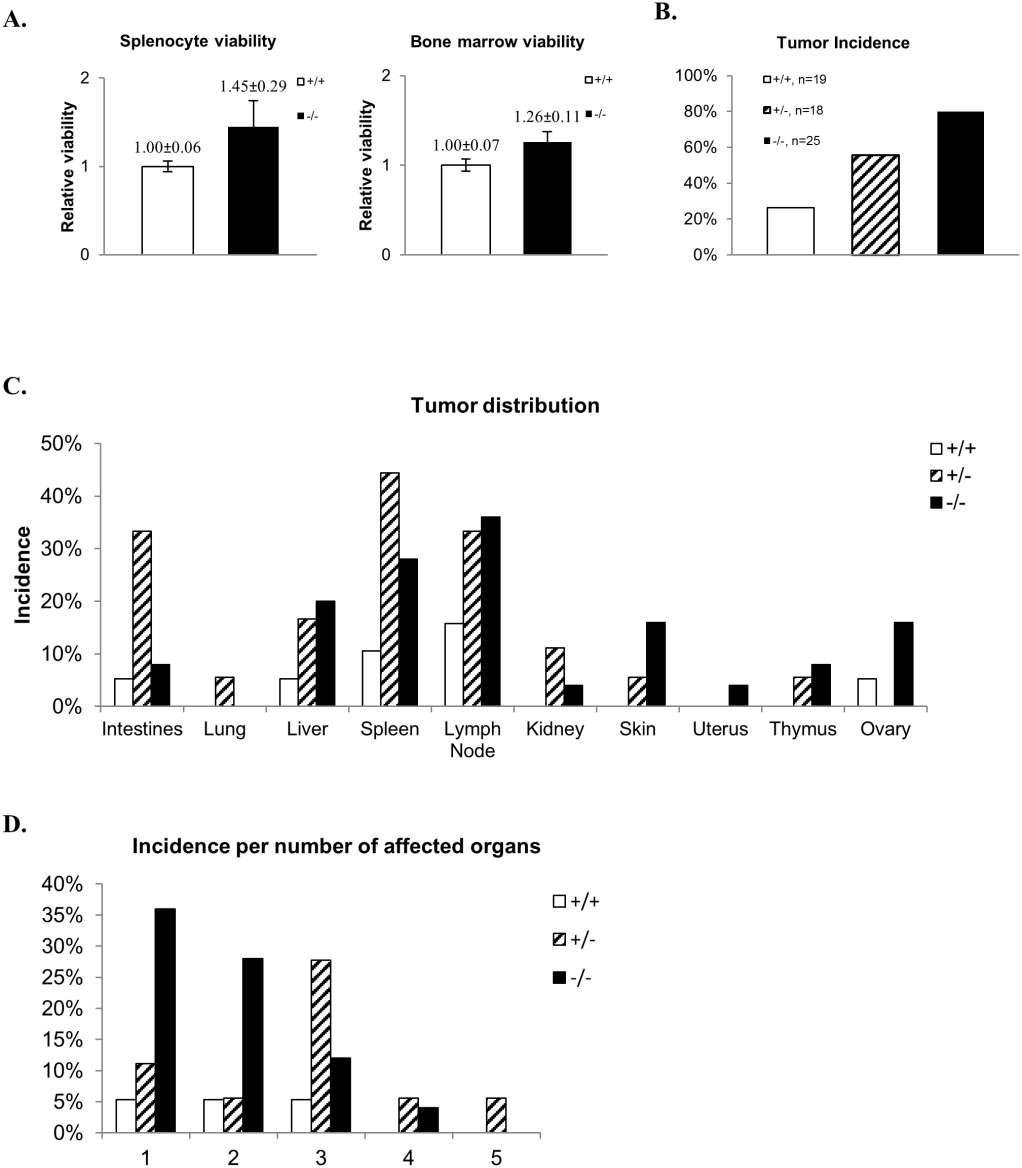
*Chc1L* ^+/-^ and *Chc1L* ^-/-^ mice have elevated tumor incidence. A) Splenocyte and bone marrow cell viability. *Chc1L* ^-/-^ splenocytes and bone marrow cells have increased viability following LPS-stimulation, compared wild-type controls (splenocyte fold-survival *Chc1L* ^-/-^/ *Chc1L* ^+/+^ = 1.45±0.29, p<0.05, n = 3; bone marrow fold-survival *Chc1L* ^-/-^/*Chc1L* ^+/+^ = 1.26±0.11, p<0.01, n = 3). B) Tumor incidence. Tumor incidence by genotype. Incidence of observable tumors was highest in *Chc1L* ^+/-^ and *Chc1L* ^-/-^ mice (*Chc1L* ^+/+^: 26%, *Chc1L* ^+/-^: 56%, *Chc1L* ^-/-^: 80%). C) Tumor distribution. Tumors were found most often in the spleen, mesenteric lymph nodes and liver. D) Incidence of multiple organs being affected. *Chc1L* ^+/-^ mice typically had multiple tumor-bearing organs.

However, as our initial interests were MM and CLL, when spleen and bone marrow cell suspensions from 8–12 week old mice were cultured in the presence of lipopolysaccharide (LPS), *Chc1L* ^-/-^ cells had a consistently increased survival rate compared to controls (Fig 2A).

Allowing for neoplastic progression, mice were sacrificed at 18–26 months, and detailed necropsies were performed. Tumors were significantly more prevalent in *Chc1L* ^+/-^ (10/18) and *Chc1L* ^-/-^ (20/25) mice, compared to *Chc1L* ^+/+^ controls (5/19), p<0.01 (Fig 2B). Tumors were most commonly observed in the spleen (*Chc1L* ^+/-^: 8/18; *Chc1L* ^-/-^: 7/25), mesenteric lymph nodes (*Chc1L* ^+/-^: 6/18; *Chc1L* ^-/-^: 9/25), liver (*Chc1L* ^+/-^: 3/18; *Chc1L* ^-/-^: 5/25), and intestines (*Chc1L* ^+/-^: 6/18; *Chc1L* ^-/-^: 2/25) (Fig 2C). Although overall tumor incidence in *Chc1L* ^+/-^ mice was lower than in *Chc1L* ^-/-^ mice, individual *Chc1L* ^+/-^ mice had a greater number of organs with tumors (Fig 2D). There was no indication of PC development.

### *Chc1L* disruption leads to histiocyte-rich neoplasms

**Fig 3:**
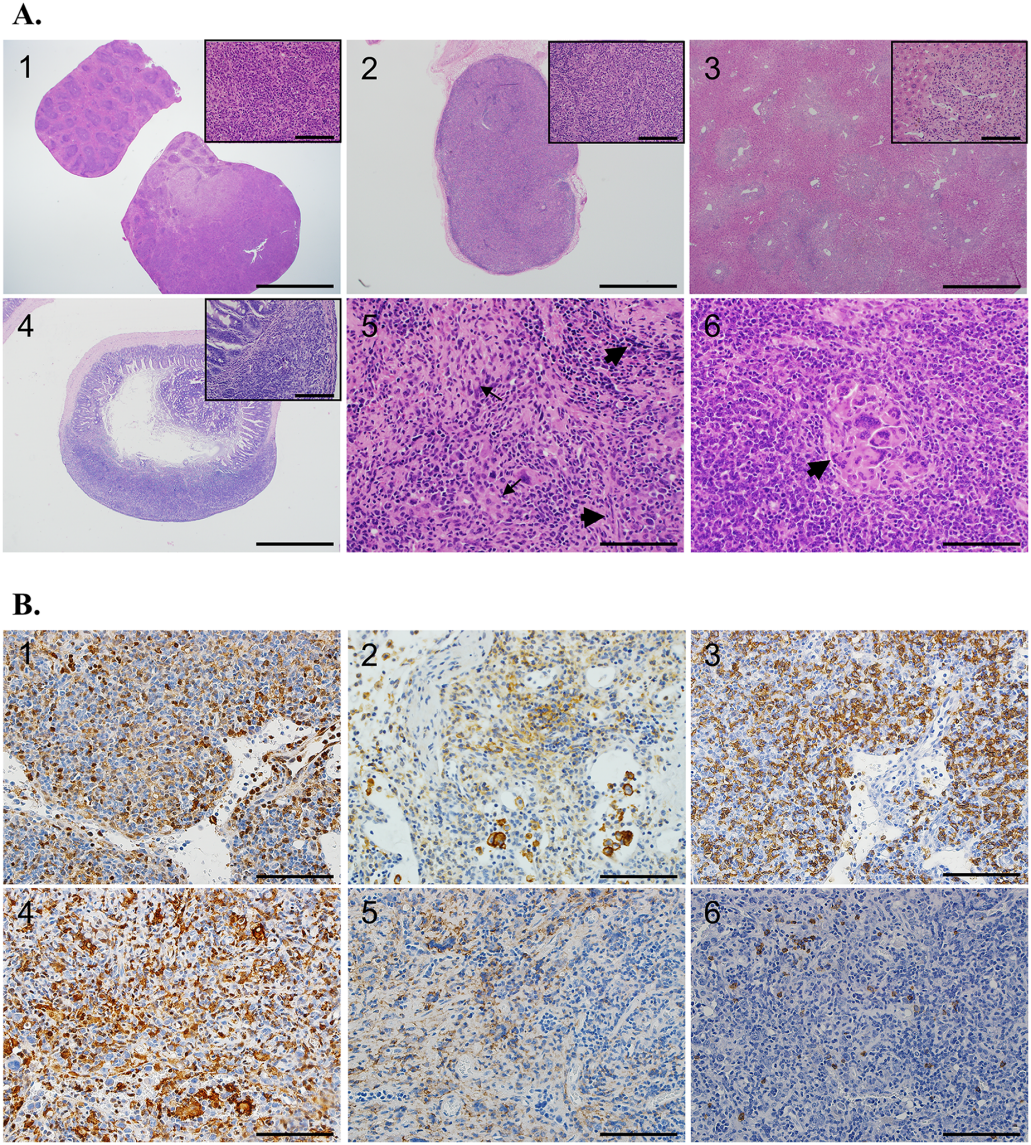
*Chc1L* ^+/-^ and *Chc1L* ^-/-^ mice develop HS and HAL. A) Representative H+E staining. Panel 1) *Chc1L* ^+/-^ and *Chc1L* ^-/-^ spleens were often enlarged, with normal structure obliterated by proliferation of tumor cells with abundant, eosinophilic cytoplasm, and irregular nuclei with open chromatin and prominent nucleoli (scale bars are 4 mm and 50 μm). Panel 2) *Chc1L* ^+/-^ and *Chc1L* ^-/-^ lymph nodes were also enlarged, with normal structure displaced by tumor cells with morphology as described in the spleen (scale bars are 1 mm and 50 μm). Panel 3) Frequently, multifocal areas of tumor cell infiltration that destroy the hepatic parenchyma were observed in *Chc1L* ^+/-^ and *Chc1L* ^-/-^ mice (scale bars are 1 mm and 50 μm). Panel 4) Peyer’s patch is severely enlarged by tumor cells which have destroyed the submucosa (scale bars are 1 mm and 50 μm). Panel 5) HS cells have pleiomorphic morphology, varying from spindle shaped (wide arrows) to round (thin arrows) (scale bar is 100 μm). Panel 6) Multinucleated giant cells (arrow) have collected in this proliferation of HS cells. Mitotic figures are abundant (scale bar is 100 μm). See S1 Fig for H+E controls. B) Immunohistochemical staining confirms diagnosis. Panels 1–3) A lymph node differentially diagnosed with HS co-occurring with B cell lymphoma or HAL. The abnormally structured lymph node is shown, with accumulation of Mac2+ (panel 1), F4/80+ (panel 2) histiocytes admixed with B220+ (panel 3) B lymphocytes (scale bars are 50 μm). Panels 4–6) A lymph node diagnosed with HS is shown. The enlarged lymph node has abundant Mac2+ (panel 4) and F4/80+ (panel 5) histiocytes, and only the occasional B220+ (panel 6) B cell (scale bars are 50 μm).

Conventional histopathological studies were performed on H+E-stained tumor sections. Representative tumors from affected tissues (spleen, lymph node, liver) were collected from *Chc1L* ^+/-^ (n = 8) and *Chc1L* ^-/-^ (n = 11) mice and compared to the same tissues of age- and sex-matched *Chc1L* ^+/+^ mice (n = 4). Spleen and mesenteric lymph nodes were frequently enlarged due to proliferation of transformed cells, expanding the tissues and destroying normal architecture (Fig 3A, panels 1 and 2) (see S1 Fig for H+E controls). The liver was often involved, and many had proliferations of neoplastic cells surrounding hepatic blood vessels (Fig 3A, panel 3). Tumors found on the lower gastrointestinal tract often caused destruction of the mucosal surface (Fig 3A, panel 4).

The morphology of the tumor cells in question varies from round to spindle-shaped, with abundant eosinophilic cytoplasm and pleiomorphic (round, oval and folded) nuclei (Fig 3A, panel 5). These features are consistent with the morphology of malignant histiocytes [14].

Often, large numbers of lymphocytes were found in the histiocyte-rich neoplasms. Mott cells, plasma cells defective in immunoglobulin secretion, that are sometimes found in MM [15], were found associated with the lymphocytes in one *Chc1L* ^+/-^ mouse. Additionally, two other *Chc1L*-deficient mice had tumor cells with a plasma cell appearance, which may indicate that myeloma-like features are a component of the disease spectrum in *Chc1L*-disrupted mice. One *Chc1L* ^-/-^ mouse examined had large numbers of multinucleated giant cells admixed with the tumor cells (Fig 3A, panel 6). Extramedullary hematopoiesis was observed in the livers and/or spleens of three mice. Abundant mitotic figures and apoptotic bodies were common.

Collectively, these findings are indicative of HS. Some tumors contain both histiocytes and large numbers of lymphocytes, and were differentially diagnosed as Histiocyte-Associated Lymphoma (HAL) or B cell lymphoma (BCL) co-occurring with HS. Table 1 summarizes the histological findings in tissues collected for analysis.

**Table 1:** Summary of histopathological diagnoses. Chc1L+/- and Chc1L-/- mice developed HS and HAL or HS co-occurring with BCL. Chc1L-/- mice developed more cases of HS, while Chc1L+/- mice had more diagnoses of HAL/HS+BCL than HS.

	+/+	+/-	-/-
**n**	19	18	25
**Cases with observed tumors**	5	10	20
**Mice examined by histopathology**	4	8	11
**Diagnoses of HS**	0	2	6
**Diagnoses of HAL/HS+BCL**	1	5	3
**Other diagnoses**	0	1	1
**No significant findings**	3	0	1

Immunohistochemistry (IHC) for histiocyte markers Mac2 and F4/80 and B cell marker B220 was performed on representative tumors to confirm these diagnoses. As shown in Fig 3B, a lymph node with HAL/HS+BCL contained cells stained positively for Mac2, F4/80 admixed with cells stained positively for B220. A separate lymph node with HS possessed Mac2 and F4/80 positive cells, which had displaced the native B220 positive lymphocytes of the tissue.

## Discussion

*CHC1L* is a candidate tumor suppressor gene located at human chromosome 13q14, a region frequently deleted in PC, MM and CLL. It is frequently underexpressed in MM and PC. However, there have been no studies confirming its tumor suppressive effect. In the present study, we provide the first direct evidence of its role in tumor suppression.

Employing Cre-Lox recombination, we generated a novel, non-conditional knockout mouse model for *Chc1L*. *Chc1L* ^-/-^ mice are born at expected Mendelian frequencies, and develop normally. At 8–12 weeks of age, cultured bone marrow and spleen isolates from *Chc1L* ^-/-^ mice have increased survival in response to LPS, compared to *Chc1L* ^+/+^ controls. We believe that this represents a heightened ability to proliferate, which, when compounded by additional mutations as the mice age, promotes tumorigenesis.

Indeed, at two years of age, both *Chc1L* ^+/-^ and *Chc1L* ^-/-^ mice were found to have a significantly higher incidence of grossly observable tumors compared to wild-type. Histopathological and immunohistochemical analyses determined that these mice were developing HS and HAL, or a composite of HS and BCL. There are presently no methods to distinguish HAL from a composite of HS and BCL [14]. Other murine models of HS have been found to possess an elevated incidence of BCL as a component of the tumor spectrum [16–18], suggesting that our mice with HAL/HS+BCL may actually have the latter diagnosis of simultaneous HS and BCL.

Histiocytic Sarcoma is a rare hematopoietic neoplasm, representing <1% of all human non-Hodgkin’s lymphomas [19]. Pathogenesis of human and murine HS is not well understood. In humans, there is evidence of HS developing from a B cell precursor, which trans- or de-differentiates into a malignant histiocyte. HS cells from patients with previous or co-occurring diagnosis of BCL have been found to contain lymphoma-specific genetic traits, including *t*(14:18), the genetic hallmark of Follicular Lymphoma (FL), and immunoglobulin rearrangements with common breakpoints between paired HS and FL tumors [20]. Several HS cases involving normal B cell genotypes have been detected [21–23]. Similarly, B cell immunophenotypes have been detected on human HS cells [24], including those diagnosed in the absence of BCL [25].

This phenomenon may occur in mice as well [26]; [14], although there is also evidence that murine HS cells arise from lymphocyte-independent pathways in the liver, stemming from Kupffer cells [14] or Ly-6C+ macrophages that develop during EMH of the liver [27]. While the cellular pathway of HS development is not known, the liver is a strong candidate primary site of pathogenesis, as it is the most commonly affected organ in murine HS [28]. We found that the liver was a common site for tumors, although less common than the spleen and lymph nodes of the mesentery.

Few studies have identified genes involved in HS development [16, 29–31], however a recent forward genetics approach has identified several candidate genes altered in murine HS [32]. Canine HS studies have determined several large cytogenetic changes involved, including frequent deletions at dog chromosome 22q11, the homologous location of human chromosome 13q14, suggesting that genes in this region are implicated in canine HS development [33]. Our findings support that *Chc1L* may be a 22q11 target gene in canine HS.

Since *CHC1L* deletion likely occurs as one component in an array of mutations that develop during tumorigenesis, future studies should focus on elucidating the relationship, including possible synergy, between *Chc1L* mutation and mutation of other genes involved in cancers affected by 13q14 deletion. Other investigations may focus on extending our findings to the bedside with clinical data supportive of *CHC1L*’s role in human HS, and in understanding *CHC1L’s* molecular pathway in order to provide insight into potentially novel therapeutic targets. CHC1L is highly homologous to RCC1, the regulator of RanGTPase. CHC1L possesses two BTB/POZ domains, protein-protein interaction domains found on E3 ligase substrate adaptors. RCBTB1, CHC1L’s paralogue, interacts with CUL3 *in vitro*, a component of E3 ligase complexes, as well as with CHC1L itself [34]. Therefore, it is possible that CHC1L may have a role as an adaptor protein, dimerizing with RCBTB1 to regulate RanGTPase via ubiquitin-mediated degradation. Changes in RanGTPase regulation may alter nuclear trafficking and chromosome condensation, resulting in abnormal proliferation and chromosomal instability [35].

In summary, this mouse model sheds light on the function of an uncharacterized gene, providing the first direct evidence for its role as a tumor suppressor gene. Further investigation will focus confirming *CHC1L*’s role in human HS and determining its molecular pathway, with the aim of identifying therapeutically targetable pathways. Additionally, by generating *Chc1L*-conditional knockout lines, our novel mouse line may be used in future studies of HS pathogenesis, particularly with respect to literature demonstrating a putative B cell precursor to HS.

## Supporting Information

S1 Fig Age-matched wild-type H+E controls. Panel 1) Spleen displaying normal architecture. Some extramedullary hematopoiesis is seen, a common finding in older mice (scale bars are 1 mm and 50 μm). Panel 2) Lymph node with normal architecture, surrounded by mesentery (scale bars are 1 mm and 50 μm). Panel 3) Liver with normal architecture. Mild inflammation is seen in the upper left quadrant, a common finding in older mice (scale bars are 1 mm and 50 μm). Panel 4) Cross-section of ileum with normal architecture (scale bars are 1 mm and 50 μm).
(TIF)

S1 File ARRIVE Checklist. Submitted as per PLOS ONE guidelines.
(DOCX)
