# Supplementary figures and images for: Chromosome Condensation 1-Like (Chc1L) Is a Novel Tumor Suppressor Involved in Development of Histiocyte-Rich Neoplasms
Source: PLoS One. 2015 Aug 20;10(8):e0135755. doi: 10.1371/journal.pone.0135755 (PMC4546397; doi:10.1371/journal.pone.0135755)

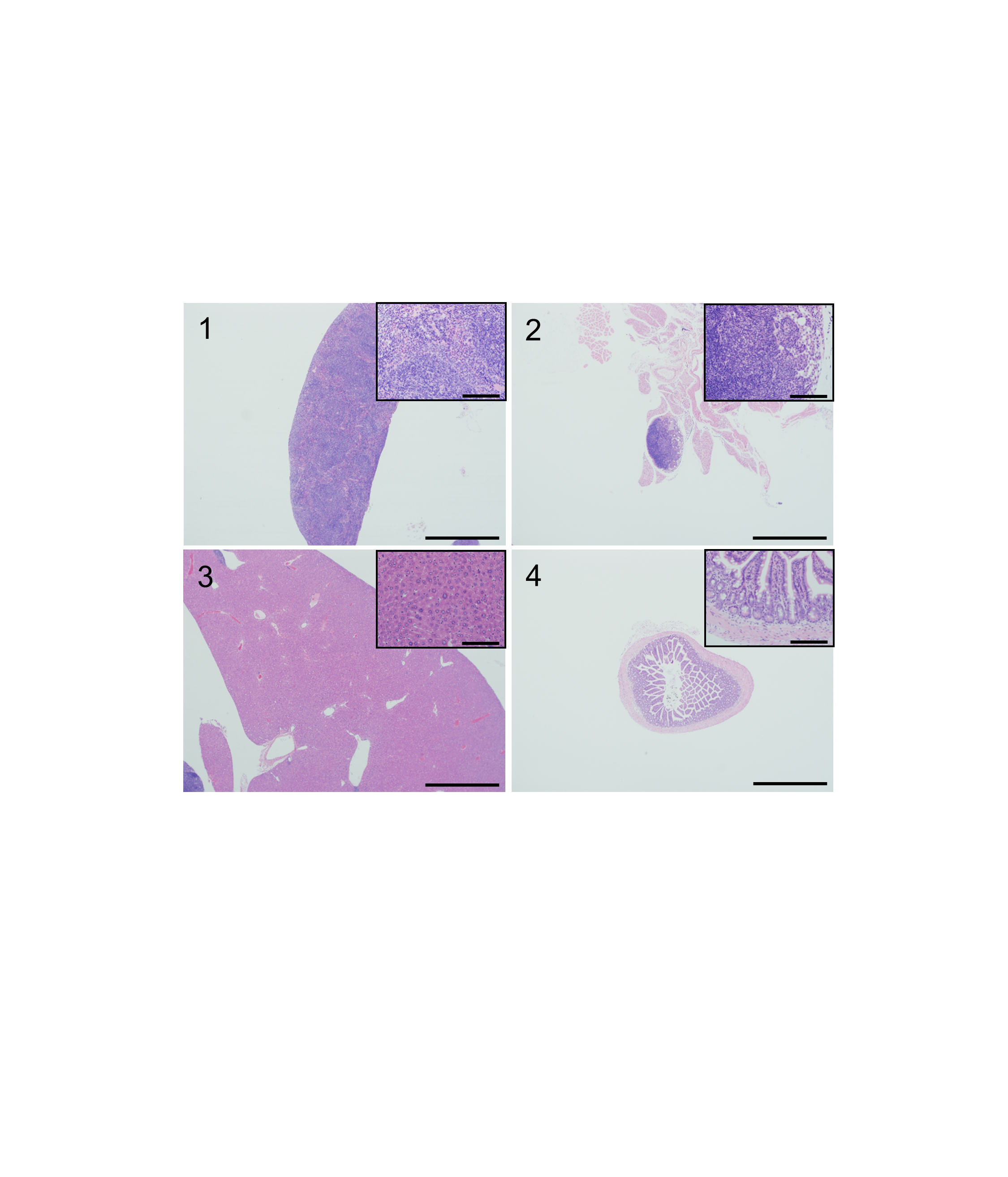

Supplement: S1 Fig — Panel 1) Spleen displaying normal architecture. Some extramedullary hematopoiesis is seen, a common finding in older mice (scale bars are 1 mm and 50 μm). Panel 2) Lymph node with normal architecture, surrounded by mesentery (scale bars are 1 mm and 50 μm). Panel 3) Liver with normal architecture. Mild inflammation is seen in the upper left quadrant, a common finding in older mice (scale bars are 1 mm and 50 μm). Panel 4) Cross-section of ileum with normal architecture (scale bars are 1 mm and 50 μm). (TIF) [file pone.0135755.s001.tif]
