# Supplementary material for: Chromosome Condensation 1-Like (Chc1L) Is a Novel Tumor Suppressor Involved in Development of Histiocyte-Rich Neoplasms
Source: PLoS One. 2015 Aug 20;10(8):e0135755. doi: 10.1371/journal.pone.0135755 (PMC4546397; doi:10.1371/journal.pone.0135755)

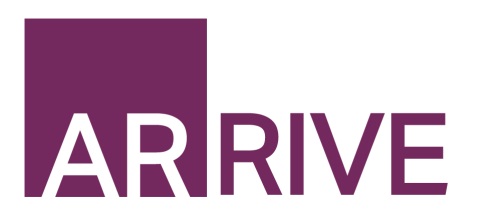


The ARRIVE Guidelines Checklist

Animal Research: Reporting In Vivo Experiments

Carol Kilkenny^1^, William J Browne^2^, Innes C Cuthill^3^, Michael Emerson^4^ and Douglas G Altman^5^

*^1^The National Centre for the Replacement, Refinement and Reduction of Animals in Research, London, UK, ^2^School of Veterinary Science, University of Bristol, Bristol, UK, ^3^School of Biological Sciences, University of Bristol, Bristol, UK, ^4^National Heart and Lung Institute, Imperial College London, UK, ^5^Centre for Statistics in Medicine, University of Oxford, Oxford, UK.*

|  | | ITEM | RECOMMENDATION | Section/ Paragraph |
| --- | --- | --- | --- | --- |
| 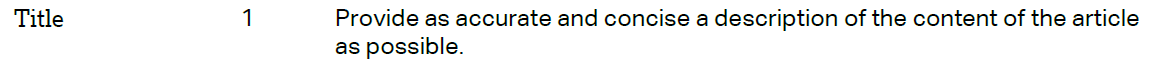 | | | Page 1 |  |
| 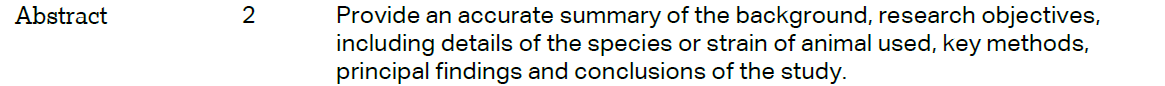 | | | Page 2: abstract |  |
| INTRODUCTION | | |  |  |
| 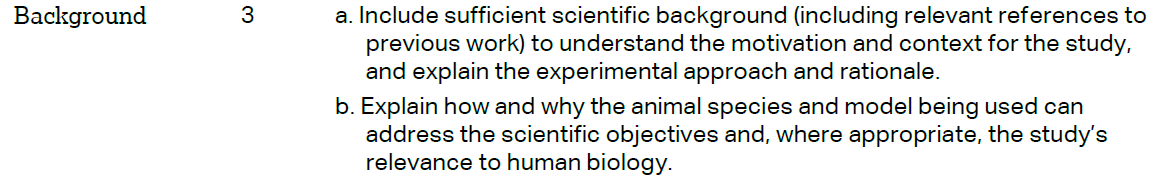 | | | Introduction: para. 1 and 2  Intro: para. 4 |  |
| 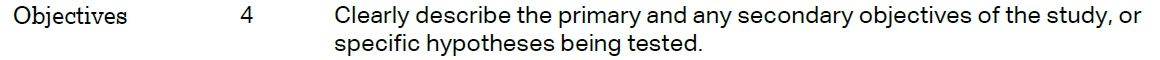 | | | Intro: para. 4 |  |
| METHODS | | |  |  |
| 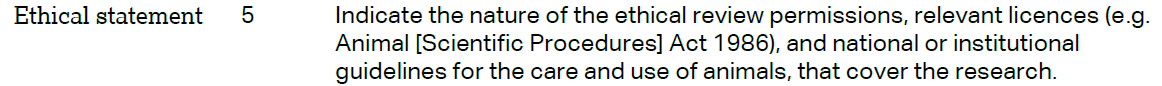 | | | Materials and methods (M+Ms): Experimental animals |  |
| 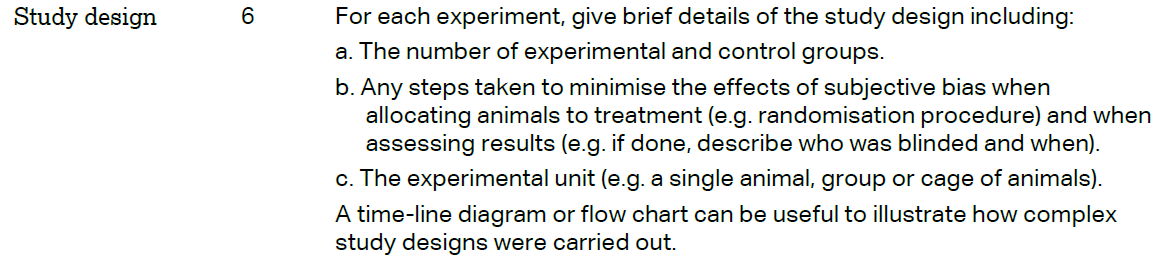 | | | See corresponding M+Ms and figure legends. |  |
| 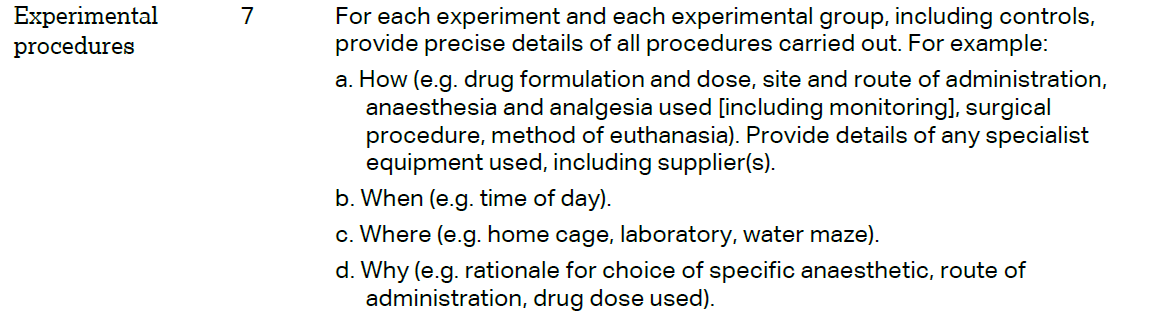 | | | Genotyping: M+Ms – Genotyping strategy  RT-PCR: M+Ms – RT-PCR  Dissections: M+Ms- experimental animals.  MTT assay: M+Ms – MTT assay  Histology: M+Ms -Histology |  |
| 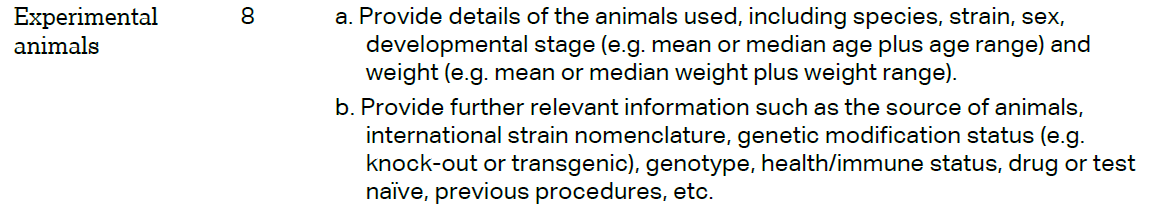 | | | M+Ms - Experimental animals. |  |

The ARRIVE guidelines. Originally published in *PLoS Biology*, June 2010^1^

| 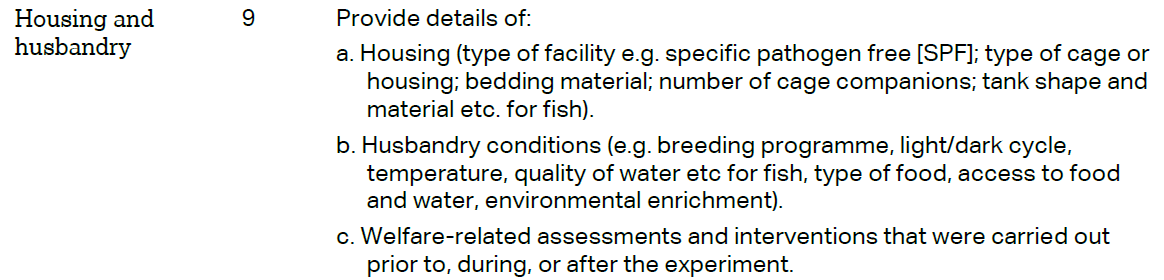 | M+Ms – Experimental animals | |
| --- | --- | --- |
| 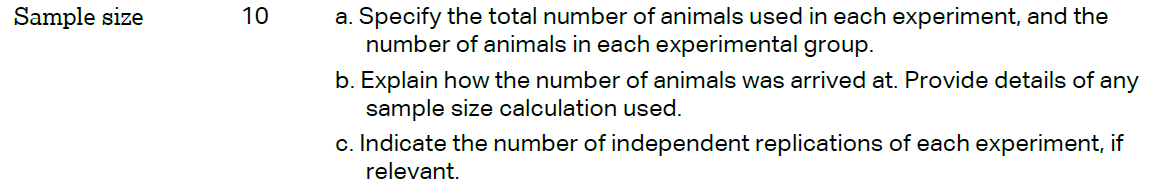 | Results – paragraphs 3 and 4 | |
| 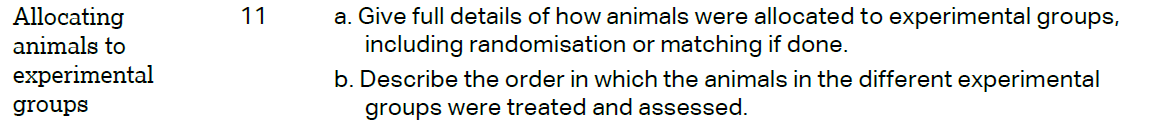 | N/A. Animals were assigned to groups based on genotype. | |
| 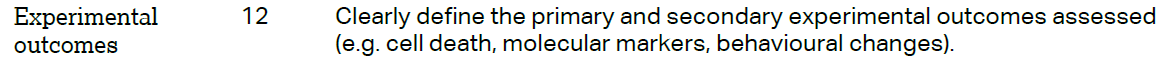 | Introduction, paragraph 4 | |
| 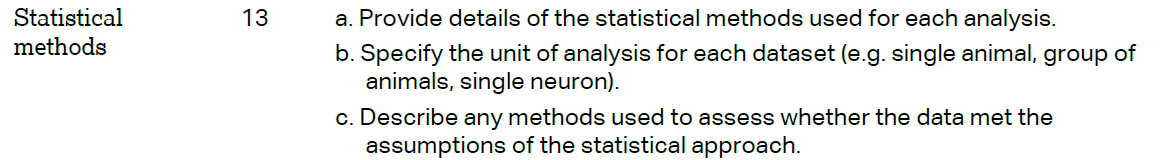 | M+Ms – Statistical methods | |
| RESULTS |  | |
| 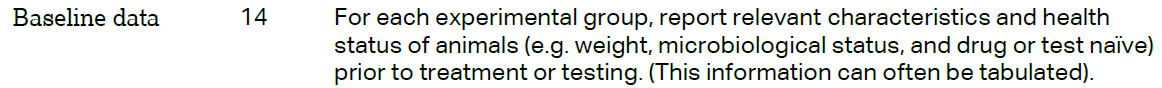 | N/A. Animals did not receive treatment nor testing | |
| 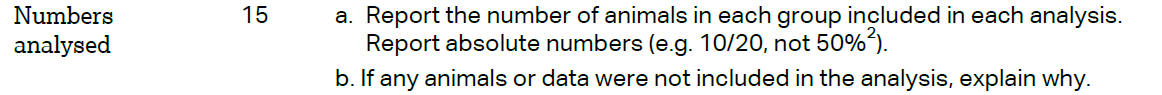 | Table 1 | |
| 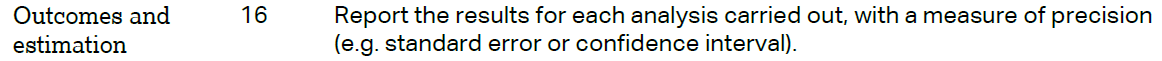 | See figures and corresponding legends | |
| 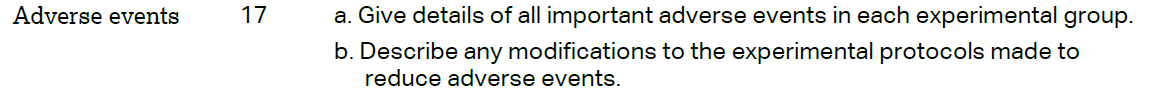 | N/A. No adverse events | |
| DISCUSSION |  | |
| 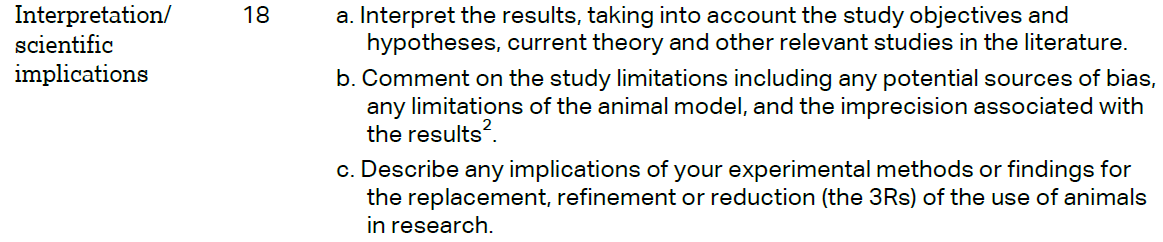 | Discussion, para. 2 and 8 | |
| 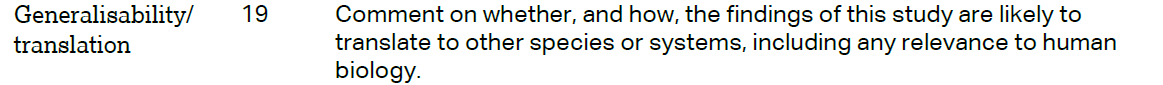 | Discussion, para. 8 | |
| 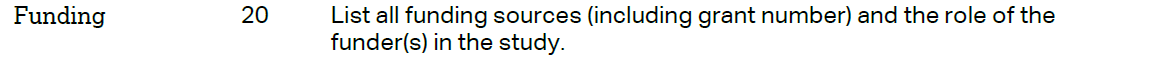 | | Acknowledgements |


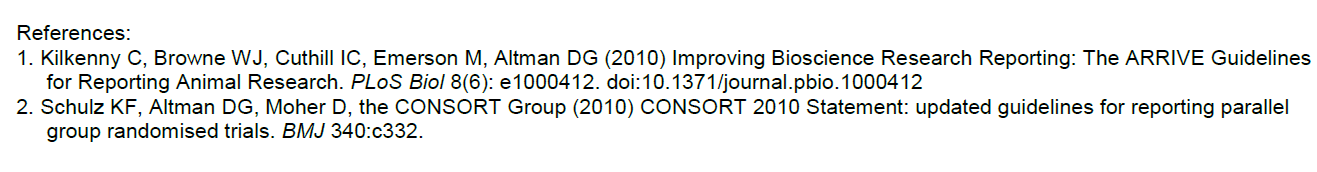

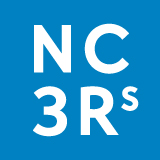

Supplement: S1 File — Submitted as per PLOS ONE guidelines. (DOCX) [file pone.0135755.s002.docx]
